# Application of *in vitro* new approach methodologies data to chemical risk assessment: current status and perspectives toward next generation risk assessment

**DOI:** 10.3389/ftox.2026.1754231

**Published:** 2026-03-18

**Authors:** Donghyeon Kim, Jinhee Choi

**Affiliations:** 1 School of Environmental Engineering, University of Seoul, Seoul, Republic of Korea; 2 Université Paris-Cité, Inserm, HealthFex, SysTox Team, Paris, France

**Keywords:** adverse outcome pathway, high-throughput toxicokinetics, *in vitro* to in vivoextrapolation, new approach methodologies, next generation risk assessment

## Abstract

The rapid transition toward animal-free chemical safety evaluation has positioned *in vitro* new approach methodologies (NAMs) as central components of next-generation risk assessment (NGRA). Advances in complex *in vitro* systems, high-content phenotypic profiling, multi-omics technologies, and AI-assisted analytics have greatly expanded the capacity to characterize human-relevant biological responses. However, despite their scientific promise, the translation of NAM-derived information into regulatory decision-making remains challenging in general. Key bottlenecks include incomplete alignment with apical regulatory endpoints, limited toxicokinetic context in conventional *in vitro* systems, and substantial variability across assays, data structures, and analytical pipelines. This review aims to summarize the current state of *in vitro* NAM technologies, evaluate the major barriers limiting their regulatory application, and discuss emerging frameworks that enable their integration into NGRA. To strengthen regulatory relevance, increasing efforts focus on integrating mechanistic NAM outputs into adverse outcome pathway (AOP) frameworks and applying high-throughput toxicokinetic (HTTK) modeling to support *in vitro*–to–*in vivo* extrapolation (IVIVE). Early NGRA case studies show that NAM-based points of departure can, in some instances, approximate or bracket traditional *in vivo* thresholds, although results remain heterogeneous across chemical classes and endpoint domains. Going forward, progress in NAM-based risk assessment will depend not only on advancements in assay technologies but also on decision frameworks capable of effectively incorporating existing NAM evidence. Tiered and evidence-integrated approaches will be essential, particularly in light of the varied NAM data availability across chemicals. Strengthening the iterative exchange between NAM application and method development will help guide future improvements and support a more transparent, adaptive, and human-relevant assessment paradigm.

## 1 Introduction

The landscape of chemical management is undergoing a major transformation as scientific, regulatory, and societal pressures drive the transition away from traditional animal-based toxicity testing (Jadhav et al., 2025). Conventional *in vivo* studies have provided decades of valuable information, yet they remain resource-intensive, low-throughput, and often limited in their ability to predict human-relevant outcomes (Wang et al., 2025). At the same time, the growing number of chemicals in commerce and the increasing demand for rapid, mechanistically informed hazard evaluation have underscored the need for innovative approaches capable of generating efficient, reliable, and human-relevant toxicological information.

New approach methodologies (NAMs)—including advanced *in vitro* systems, high-content phenotypic profiling, omics-based technologies, and AI-enabled analytical strategies—have emerged as central tools in the next-generation of predictive toxicology (Ouedraogo et al., 2025). These technologies enable more refined interrogation of biological processes, often at a depth or scale unattainable with traditional models. Complex *in vitro* systems such as organoids and microphysiological systems (MPS) provide enhanced physiological relevance, while high-content and omics-based platforms capture cellular responses across multiple levels of biology. Complementing these experimental advances, machine learning and computational methods support data-rich assay interpretation and improve the integration of mechanistic insights into risk assessment frameworks.

Despite these advances, NAMs are developed for certain endpoints, and are not in general (except for specific endpoints, e.g., skin and eye irritation, skin sensitisation), sufficient as stand-alone replacement of animal data for addressing a specific regulatory information requirement and/or end point (e.g., endocrine activity, genotoxicity, immunotoxicity). There are significant challenges remain in translating *in vitro* NAMs data into regulatory decision-making. Key bottlenecks include uncertainties in biological relevance, limited toxicokinetic context, and a lack of harmonized testing and data interpretation guidelines. As risk assessment frameworks evolve toward mechanistic, exposure-led, and human-centric paradigms—often described under the umbrella of next-generation risk assessment (NGRA)—there is a growing need to connect NAMs-derived mechanistic information with established constructs such as adverse outcome pathways (AOPs) and *in vitro*–to–*in vivo* extrapolation (IVIVE) tools including high-throughput toxicokinetic (HTTK) modeling (Ankley et al., 2010; Edmondson et al., 2014; Ashammakhi et al., 2020; Chang et al., 2022; Yang et al., 2023; Srivastava et al., 2024).

This review provides an overview of the current state of *in vitro* NAMs, examines existing barriers that hinder their use in chemical risk assessment, and discusses emerging strategies that enhance the regulatory applicability of NAM-derived data. We highlight advances in mechanistic integration through the AOP framework, developments in IVIVE and HTTK modeling, and ongoing efforts to harmonize *in vitro* testing guidelines across regulatory bodies. Case studies of NGRA applications are included to illustrate implementations of NAMs-driven decision-making. Finally, we present remaining limitations and future perspectives to support the continued evolution of NAMs toward a scientifically robust and broadly accepted foundation for next-generation risk assessment.

## 2 State-of-the-art *in vitro* NAMs

**FIGURE 1:**
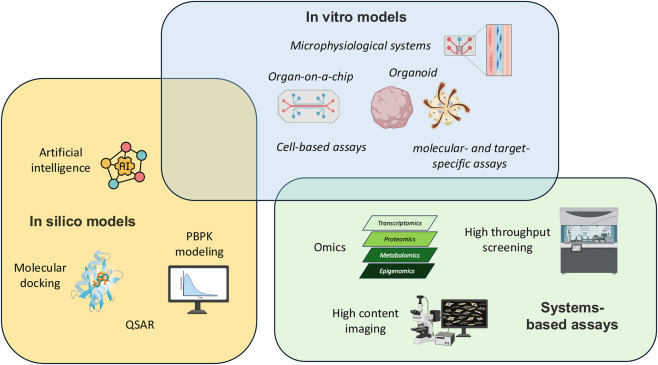
The landscape of state-of-the-art new approach methodologies (NAMs).

Recent advances in NAMs have diversified the landscape of *in vitro* approaches used for human-relevant chemical safety assessment. Classical *in vitro* testing has evolved beyond traditional cell-based assays and is increasingly integrated with high-throughput and high-content screening platforms, omics-driven molecular profiling, and data-driven computational tools such as AI. At the same time, the development of advanced *in vitro* models—including 3D cultures, organoids, and microphysiological systems—has expanded the capacity to capture more physiologically meaningful responses. As illustrated in Figure 1, current *in vitro* NAMs span multiple technological domains, ranging from molecular- and target-specific assays to complex tissue-mimetic systems, and from experimental platforms to computational frameworks.

### 2.1 Advanced *in vitro* models

Advanced *in vitro* models have progressively evolved beyond traditional two-dimensional cell cultures to provide enhanced physiological relevance for human health risk assessment. Three-dimensional cultures, organoids, organ-on-a-chip platforms, and multi-organ microphysiological systems (MPS) recapitulate key features of tissue and organ function, including cell–cell and cell–matrix interactions, metabolic competence, barrier integrity, and coordinated tissue-level responses (Edmondson et al., 2014; Ashammakhi et al., 2020; Yang et al., 2023; Srivastava et al., 2024). These systems enable the evaluation of complex biological processes such as chronic or repeated exposures, organ-specific toxicity, and inter-organ communication that are not readily captured by simpler models. Despite their promise, widespread application remains limited by challenges including technical complexity, variability between platforms, limited throughput, and a lack of standardized protocols. Continued efforts to improve reproducibility and validate system performance will be critical for increasing their regulatory acceptance and integration into next-generation risk assessment frameworks.

### 2.2 High-content phenotypic profiling

High-content phenotypic profiling (HCP) encompasses image-based assays that quantify morphological and functional cellular changes across hundreds of features simultaneously (Hughes et al., 2021). Techniques such as high-content imaging (HCI), Cell Painting, and multiplexed reporter assays generate rich phenotypic signatures that reflect diverse cellular responses to chemical perturbation (Bray et al., 2016; Way et al., 2023). Because HCP captures a wide spectrum of morphological and subcellular alterations without reliance on predefined endpoints, it enables unbiased detection of early cellular stress responses and provides a sensitive platform for characterizing chemical-induced perturbations. These multidimensional profiles can be used to classify chemicals based on phenotypic similarity, explore potential modes of action, and support read-across or prioritization activities in chemical evaluation (Grimm et al., 2019). Increasingly, artificial intelligence and machine learning approaches are incorporated into HCP pipelines to enhance feature extraction, pattern recognition, and assay reproducibility, enabling higher scalability and more robust phenotypic interpretation.

### 2.3 Omics technologies

Omics-based NAMs capture chemical-induced biological responses at the molecular level, providing comprehensive insight into transcriptional, proteomic, metabolic, and epigenetic alterations (Chen et al., 2023). High-throughput transcriptomic approaches such as RNA-seq and TempO-Seq enable sensitive detection of early gene expression changes, while proteomics and metabolomics capture downstream effects on cellular pathways, metabolic flux, and functional biochemical processes (Wang et al., 2009; Wehr et al., 2024). These molecular signatures support the identification of perturbed biological pathways, network-level responses, and potential molecular initiating events (MIEs). Omics data therefore represent a critical foundation for mechanistic toxicology.

In addition to mechanistic insights, omics data have increasingly been used to derive quantitative points of departure (PODs), including transcriptomic benchmark dose values and NO(A)EL-equivalent estimates generated through concentration–response modeling of gene expression profiles (Johnson et al., 2022). Such quantitative applications of omics technologies aimed to integrate omics data into risk assessment. A prominent example of this trend is the EPA Transcriptomic Assessment Product (ETAP) framework developed by the U.S. Environmental Protection Agency, which uses transcriptomic data to determine a reference daily dose for chemicals lacking traditional toxicity testing data (U.S. Environmental Protection Agency, 2023). ETAPs leverage changes in gene activity measured in short-term studies to derive transcriptomic PODs and corresponding reference values that can be produced in a matter of months, thereby facilitating more timely health assessments compared with traditional chronic testing approaches.

However, the regulatory integration of omics data remains limited by platform-dependent variability, data interpretation complexity, and a lack of harmonized analytical and reporting frameworks. Standardization of data generation, quality control metrics, and reference datasets will be essential for expanding the utility of omics technologies in regulatory decision-making (Chervitz et al., 2011).

### 2.4 AI-assisted strategies

Artificial intelligence (AI) and machine learning (ML) are rapidly emerging as integral components of *in vitro* NAMs, providing enhanced analytical, predictive, and integrative capabilities across diverse data types (Sun et al., 2025). AI-assisted strategies support multiple stages of the NAM workflow, from experimental design and quality control to data processing, feature extraction, and mechanistic interpretation. In image-based assays such as high-content phenotypic profiling, deep learning models facilitate automated segmentation, pattern recognition, and the identification of subtle phenotypic perturbations that may be challenging to detect using traditional analytical approaches (Chandrasekaran et al., 2021). Similarly, in omics-based NAMs, ML algorithms enable dimensionality reduction, clustering of exposure signatures, and the inference of perturbed pathways or molecular initiating events (Tabakhi et al., 2023). Beyond single-platform applications, AI plays a pivotal role in integrating heterogeneous data generated from advanced *in vitro* models, phenotypic assays, and multi-omics technologies. These integrative models can support cross-platform harmonization, enhance confidence in mechanistic predictions, and help establish quantitative relationships between molecular, cellular, and tissue-level responses (Way et al., 2023). AI-based modeling approaches are also increasingly applied to read-across, potency ranking, and risk-relevant predictions, accelerating the transition toward data-driven, non-animal NGRA frameworks.

## 3 Current status of regulatory uptake of *in vitro* NAMs for chemical risk assessment

Over the past years, *in vitro* NAMs have achieved meaningful regulatory uptake for several local toxicity endpoints, particularly within well-defined adverse outcome pathways (AOPs). Notable progress has been made in the areas of skin sensitization, skin corrosion/irritation, and eye irritation, where integrated approaches to testing and assessment (IATAs) and defined approaches incorporating validated *in vitro* assays have been formally adopted by multiple regulatory authorities. For example, the OECD has established test guidelines for key *in vitro* assays such as DPRA, KeratinoSens, and h-CLAT for skin sensitization, and for reconstructed human epidermis and cornea models for irritation and corrosion. These test guidelines can be accepted in regulatory submissions as either partial replacements or full alternatives to traditional animal testing. Despite these advances, the extent to which such OECD-validated NAMs are utilized in regulatory decision-making varies considerably across jurisdictions. For instance, in Korea, although NAM-based OECD test guidelines are formally recognized within the national regulatory framework, their practical use in chemical registration and evaluation processes remains extremely limited. Beyond these local endpoints, however, the regulatory uptake of *in vitro* NAMs remains constrained for systemic toxicity domains such as repeated-dose toxicity, carcinogenicity, developmental toxicity, and neurotoxicity. Consequently, current regulatory use of NAMs in these areas is exploratory or supportive.

## 4 Main bottlenecks in applying *in vitro* NAMs for chemical risk assessment

Despite the rapid advances in *in vitro* NAM technologies, several key barriers still limit their full integration into regulatory chemical risk assessment. In this study, we focused on three major bottlenecks that continue to hinder broader implementation: (1) limited relevance of NAM outputs to traditional regulatory endpoints, (2) lack of toxicokinetic context, and (3) limited harmonization across assays and platforms. These issues do not diminish the scientific value of NAMs but highlight areas where further development, standardization, and integration with complementary tools are needed.

### 4.1 Lack of relevance of *in vitro* data to regulatory endpoints

Regulatory agencies typically base hazard determinations on apical outcomes observed at the whole-organism level—such as organ toxicity, carcinogenicity, reproductive toxicity, and developmental effects. In contrast, many of the NAMs described in Section 2 focus on upstream mechanistic signals, including pathway activation, cellular stress responses, or morphological alterations. Although these early biological perturbations are scientifically meaningful, they do not always clearly indicate whether an adverse health effect will emerge *in vivo.*

For example, an *in vitro* hepatocyte model may reveal steatosis or mitochondrial dysfunction, but such findings alone do not establish whether these perturbations would progress to clinically meaningful liver injury in humans (Bedossa, 2017; Allard et al., 2021). Even advanced *in vitro* systems such as organoids and microphysiological systems (MPS), despite their improved physiological fidelity, still lack system-level integration, including real-time circulation, immune interactions, endocrine regulation, and multi-organ signaling—factors that critically influence toxicity outcomes *in vivo*.

Regulatory bodies including the U.S. FDA and OECD acknowledge that no single NAM currently captures all critical hazard endpoints across human organ systems, and consequently, NAM data are often used in supportive roles—such as providing mechanistic evidence, informing chemical prioritization, or flagging potential hazards—rather than serving as standalone replacements for all guideline-required studies (OECD, 2022; FDA, 2024). Strengthening the regulatory relevance of NAMs will require more robust mechanistic anchoring through the Adverse Outcome Pathway (AOP) framework, the development of quantitative AOPs (qAOPs), and clearer linkages between molecular or cellular perturbations and adverse outcomes (Escher et al., 2022).

Importantly, the relevance gap is not solely attributable to limitations of NAMs themselves. Increasing discussion in the toxicology and regulatory science communities suggests that part of the mismatch arises because the regulatory endpoint framework has not evolved at the same pace as modern mechanistic toxicology (Rovida et al., 2015). Traditional hazard categories—such as acute or chronic toxicity and CMR classifications—were originally defined around pathology-based observations in animal studies, with limited mechanistic specificity and only indirect connection to human biology. In contrast, contemporary NAMs are explicitly designed around human-relevant, hypothesis-driven, and target organ–specific mechanisms (Schmeisser et al., 2023; Sewell et al., 2024). As a result, NAM outputs may be biologically meaningful and closely aligned with human pathways, yet they do not map neatly onto legacy regulatory endpoints that remain anchored in broad apical outcome categories. This structural misalignment indicates that advancing NAM relevance will require not only improved mechanistic integration, but also modernization of regulatory endpoint frameworks to better reflect current biological understanding.

### 4.2 Lack of toxicokinetic context

Most *in vitro* NAM assays quantify biological activity based on nominal concentrations in culture media, yet these concentrations rarely reflect the complex toxicokinetic processes occurring *in vivo*. In a living organism, absorption, distribution, metabolism, and excretion (ADME) shape the internal dose at the site of action, whereas static *in vitro* systems lack metabolic clearance, blood flow, tissue partitioning, or protein binding dynamics (Wang et al., 2015). Although advanced *in vitro* models such as 3D cultures, organoids, and microphysiological systems (MPS) partially address these gaps by providing improved metabolic competence, more realistic tissue partitioning, and, in some cases, dynamic perfusion, they still fall short of capturing whole-body ADME processes—including systemic circulation, renal and biliary clearance, and multi-organ interactions—that ultimately determine exposure at target sites in humans. Consequently, a chemical may appear potent *in vitro* at micromolar levels that are never achieved *in vivo*, or conversely, a persistent and bioaccumulative compound may reach tissue concentrations far exceeding those tested in culture. Protein binding, lipid partitioning, and metabolite formation can further complicate interpretation, contributing to systematic overestimation or underestimation of hazard when using *in vitro* potency alone.

These limitations demonstrate that nominal concentrations cannot always serve as direct surrogate measures of *in vivo* exposure; however, several studies have shown that nominal doses can function as conservative or “worst-case” estimates when compared with actual human exposure levels (Nicol et al., 2024). Accordingly, nominal concentrations in in vitro systems may still provide a health-protective basis for screening-level hazard assessments. However, this conservative tendency can, in some cases, lead to overestimation of hazard *in vitro*, introducing uncertainty that may undermine confidence in NAM-based evaluations. Therefore, to support more reliable regulatory decision-making, it is ideal to interpret *in vitro* NAM data by considering both the limitations and conservative nature of nominal concentrations together with relevant exposure and toxicokinetic information.

In this regard, the International Cooperation on Cosmetics Regulation (ICCR) outlined the principles of the NGRA framework, emphasizing that assessments should be exposure-led (Dent et al., 2018). This principle underscores the need to interpret hazard-focused NAM data within a human exposure–relevant context. Without appropriate toxicokinetic information, NAM outputs cannot reliably indicate whether observed *in vitro* activity is expected to occur at realistic exposure levels. Therefore, generating and integrating toxicokinetic-focused NAM data alongside hazard-focused NAM outputs remains a key challenge for enabling the effective use of *in vitro* NAMs within an exposure-led NGRA framework.

Accordingly, this limitation underscores the importance of coupling *in vitro* NAM data with *in vitro*–to–*in vivo* extrapolation (IVIVE) (Chang et al., 2022), high-throughput toxicokinetic (HTTK) modeling (Pearce et al., 2017), and quantitative dosimetry tools—topics discussed in later sections.

### 4.3 Limited harmonization

A persistent barrier to widespread regulatory adoption is the lack of harmonization across NAM assays, platforms, data standards, and analytical pipelines. NAMs currently span a wide technological spectrum—from simple biochemical tests to complex organ-on-chip systems—and each system often uses distinct protocols, culture media, exposure durations, endpoints, and data processing workflows. As a result, studies assessing similar biological processes can yield outputs that vary substantially between laboratories (Stucki et al., 2022).

High-content phenotypic profiling illustrates this challenge: imaging platforms differ in staining panels, segmentation algorithms, and feature extraction methods, making cross-study comparisons difficult. Likewise, omics technologies generate vast datasets with disparate formats, metadata structures, and normalization approaches, complicating integration into regulatory evidence streams. Advanced *in vitro* models face similar issues. Organoids and microphysiological systems lack universally accepted validation criteria, and performance metrics such as functional stability or fidelity to human physiology vary across developers and users.

Regulatory toxicology requires transparent, reproducible, and well-annotated datasets, yet NAM outputs are often reported in diverse formats that lack standardized descriptors or ontologies (Ouedraogo et al., 2025). This heterogeneity limits their usability in chemical regulation. Ongoing efforts—including OECD guidance documents, community-driven minimum information standards, and emerging NAM validation frameworks—are beginning to address these issues, but adoption remains uneven across the field.

## 5 Integrating *in vitro* NAM data for mechanistic and quantitative interpretation

### 5.1 Integration of *in vitro* NAMs data into adverse outcome pathway

To improve the relevance of *in vitro* data to regulatory endpoints, adverse outcome pathway (AOP) frameworks can be used (Ankley et al., 2010). The AOP framework has emerged as a key strategy to integrate mechanistic *in vitro* data (from NAMs) with the higher-level outcomes needed for risk assessment. An AOP is essentially a conceptual map that links a Molecular Initiating Event (MIE) – often a chemical’s interaction with a biological target–through a series of intermediate Key Events (KEs) at increasing levels of biological organization, ultimately to an Adverse Outcome (AO) of regulatory concern (e.g., an organism-level effect like neurotoxicity or reproductive impairment). By providing a causal chain from molecule to disease, AOPs create a bridge between *in vitro* mechanistic findings and *in vivo* adverse effects. In this framework, each NAM test can be seen as probing a specific key event. For example, an *in vitro* assay might detect activation of a receptor (a molecular initiating event) or cell death in a particular tissue model (a key event), which the AOP then connects to a potential adverse outcome (like liver fibrosis or developmental toxicity). Integrating NAM data into AOPs means mapping the results of *in vitro* tests onto these key event nodes. If a chemical produces a positive response in a NAM that corresponds to an upstream key event in a known AOP, it serves as an early warning that the chemical could progress toward the adverse outcome down the line. For instance, if a chemical is positive in a thyroid hormone synthesis inhibition assay (an upstream key event in several developmental neurotoxicity AOPs), that indicates a risk for downstream neurodevelopmental harm, even if no whole-animal test has been done. The AOP framework thus contextualizes isolated NAM findings within a broader biological narrative, helping risk assessors interpret what a NAM result might mean for organism health. It also encourages a weight-of-evidence approach: multiple NAMs targeting different key events in the same pathway can collectively increase confidence in a predicted outcome. Importantly, AOPs are being made machine-readable and modular, via efforts like the OECD’s AOP Knowledgebase and AOP-Wiki, so that diverse data sources can be integrated and analyzed systematically. While the AOP framework is still expanding (with many AOPs in development and not all toxicities covered), it provides a promising scaffold to formalize the integration of NAM data. By anchoring *in vitro* results to mechanistic pathways leading to adverse effects, AOPs help regulators and scientists use NAM evidence in a more transparent and scientifically grounded way for risk assessment. Ultimately, as more AOPs are established and quantified, they will enable a shift from describing hazard in terms of animal observations to describing it in terms of networks of biological perturbations–a shift that is very much in line with the strengths of NAMs.

### 5.2 *In vitro* to *in vivo* extrapolation with high throughput toxicokinetic modeling

A crucial step in utilizing *in vitro* data for risk assessment is figuring out what an observed effect in a cell culture means for a whole living organism. This is the role of *in vitro* to *in vivo* extrapolation (IVIVE). IVIVE involves translating the concentration of a chemical that causes an effect in an *in vitro* test (e.g., an EC50 in a cell assay) into an equivalent *in vivo* exposure level (such as a dose in mg/kg/day) that would lead to a similar effect in an animal or human. In essence, IVIVE asks: “If this chemical is toxic to liver cells at X micromolar in a petri dish, what dose of the chemical would produce that blood concentration in a person?” This requires understanding toxicokinetics (TK) – how a chemical is absorbed, distributed, metabolized, and excreted in the body. High-throughput toxicokinetic modeling has been developed to perform IVIVE rapidly for large numbers of chemicals. For example, the U.S. EPA’s approach uses generic physiologically based pharmacokinetic (PBPK) models and *in vitro* measures of metabolism and plasma protein binding for many chemicals to estimate the administered equivalent dose (AED) that would achieve a given blood concentration. Tools like the Integrated Chemical Environment’s IVIVE dashboard automate this process, allowing researchers to input *in vitro* potency values and compute corresponding *in vivo* dose estimates using built-in TK models. High-throughput TK (HTTK) modeling can incorporate data from *in vitro* metabolic clearance assays (e.g., using human liver microsomes or hepatocytes) and computational ADME predictions to predict how quickly a chemical is eliminated, which in turn influences the blood concentrations from a given dose. By doing this for many chemicals, one can rank chemicals by their inferred *in vivo* potency even if *in vivo* studies are lacking. IVIVE is therefore a cornerstone of next-generation risk assessment (NGRA) frameworks that rely on NAMs. It enables risk assessors to derive points of departure (like no-effect levels or benchmark doses) from purely *in vitro* data. For instance, the ToxCast program has used IVIVE to estimate oral doses that would correspond to bioactive *in vitro* concentrations, facilitating comparisons to human exposure estimates (Jeong et al., 2022). Despite its power, IVIVE comes with challenges–ensuring the models are accurate, accounting for inter-individual differences, and handling chemicals with complex kinetics (e.g., bioaccumulative or rapidly metabolized compounds). Yet continuous improvements are underway: new high-throughput toxicokinetic data generation (measuring thousands of compounds’ metabolic rates and binding), AI/ML enhancements to TK predictions, and even “virtual organisms” modeling multiple pathways simultaneously. In summary, IVIVE using high-throughput TK modeling is the key that unlocks *in vitro* NAM data for quantitative risk assessment–by providing the link between a cell-based effect level and a real-world exposure scenario. This approach is increasingly being refined and validated, bringing purely *in vitro* risk assessments closer to reality.

## 6 Application of NAMs in NGRA and future directions

### 6.1 NGRA case studies: utilization of *in vitro* NAMs data

Recent applications of NGRA workflows that couple high-throughput NAM bioactivity data with HTTK-based IVIVE have shown that NAM-derived points of departure (PODs) are often more conservative and, in some cases, fall within a similar range to traditional *in vivo* PODs (Table 1). While these findings are encouraging and provide early indications of the potential utility of NAM-based approaches in risk assessment, they should be interpreted cautiously given that the evidence base is still developing.

**TABLE 1:** Summary of representative NGRA case studies.

Chemical		*In vitro* data	*In vivo* data	PBPK		*In vitro* and *in vivo* comparison result	References
Library	No.			Model	Cmax		
HC Yellow No. 13 (Hair dye)	1	In house experiments using human stem cell-derived hepatic cells (hSKP-HPC)	Not used	Compartmental model and PBPK model (GastroPlus 9.9)	Liver	• PoD_NAM values exceeded predicted Cmax levels, indicating no expected liver steatosis risk	Sepehri et al. (2025)
Benzophenone-4 (UV filter)	1	Various *in vitro* testing	Not used	Population PBPK model (Gastroplus)	Plasma, liver, kidney	• PoD_NAM values generally exceeded predicted human Cmax levels, indicating no safety concern	Maria and Baltazar (2025)
Sodium-2-hydroxyethane sulfonate	1	Various *in vitro* testing	Not used	Gastroplus PBK (15-compartment)	Plasma	• Lowest PoD_NAM values exceeded predicted Cmax levels, indicating no expected systemic or developmental toxicity risk at occupational exposure levels	Wood et al. (2024)
Benzyl salicylate	1	Various *in vitro* testing	CIR review and SCCS opinion	Probabilistic PBPK	Plasma	• NAM-based margin of internal exposure were more conservative than those derived from external exposure and *in vivo* PODs	Ebmeyer et al. (2024)
EPA comptox chemical dashboard	448	ToxCast	ToxValDB	HTTK R-package	Plasma	• Of the 448 substances, 89% had a POD_NAM,95 that was less than the traditional POD (POD _traditional) value	Paul Friedman et al. (2020)
Health Canada’s DSL (Domestic substances list)	5801	ToxCast and in silico-based predicted bioactivity	ToxValDB	HTTK R-package	Plasma	• The vast majority of chemicals (95.20%) had POD_bioactivity or POD_Read-Across values that were protective in that they were lower than or equal to POD_traditional	Beal (2021)
Bisphenol group	4	ToxCast ER agonist assays	Scientific reports from regulatory agencies	Bisphenol-specific PBPK model	Gonads	• Animal POD-derived HED estimates fell within the range of *in vitro* AC_10_-derived HEDs for BPA, BPF, BPAF, while for BPS, the range of in vitro-based HEDs could include or be close to the animal-based HEDs	Lin and Lin (2020)
A group of substituted phenols	3	EDSP212 (ToxCast) and CERAPP	*In vivo* uterotrophic assays	HTTK R-package and ACD PERCEPTA	Plasma	• The lower limit of the ER-related AED for 4-ter-butylphenol was found to be more health protective than the NOAEL from the *in vivo* two-generation reproductive toxicity study	Webster et al. (2019)
A group of 29 ER agonists	29	ToxCast ER agonist assays	*In vivo* uterotrophic assays	Three PK modeling approach	Plasma	• The BMDL10 differed only 3-fold from the reported NOAEL values	Casey et al. (2018)
Environmental chemicals potentially interact with ER pathway	230	Tox21 ER transactivation assay	*In vivo* uterotrophic assays	Simple PK equation (Linear)	Plasma	• The oral equivalent doses (OEDs) estimated from the *in vitro* POD of an ER transactivation assays were lower than the LELs in rat uterotrophic assays	Chang et al. (2015)
ToxCast chemical library and more	112	Human uterine cell estrogen response assay (IKA assay) and ToxCast assays	*In vivo* uterotrophic assays	HTTK R-package	Plasma	• A human uterine cell estrogen response assay was used to estimate *in vivo* equivalent doses for a set of chemicals and found 19 out of 23 chemicals to have an AED lower or equivalent to PODs in the rodent uterotrophic assay	Beames et al. (2020)

Across several large chemical datasets, comparable patterns have been observed. For example, Paul Friedman et al. (2020) evaluated 448 chemicals using ToxCast bioactivity data and HTTK modeling and reported that a substantial proportion of NAM-derived PODs were lower than corresponding animal-based values, with most other comparisons falling within an order of magnitude. Beal (2021), examining more than 5,800 chemicals on Canada’s Domestic Substances List, similarly found that NAM-based PODs frequently overlapped with or were more protective than traditional PODs, although variability remained across chemicals and endpoints.

Comparable trends have been reported for targeted chemical groups. Studies on bisphenols, substituted phenols, and estrogen receptor agonists—using PBPK models, HTTK analyses, or simpler PK equations—have shown that *in vitro*–derived AEDs often fall near established NOAEL, LOAEL, or uterotrophic effect levels. However, these results are restricted to specific chemical classes and experimental conditions and should not be generalized broadly to all toxicological domains or chemical structures.

Taken together, these case studies provide preliminary evidence that integrating NAM bioactivity data with IVIVE and HTTK modeling may support the derivation of health-protective PODs within NGRA frameworks. Nevertheless, these applications remain in an early stage of development, and key uncertainties persist, including model accuracy, representation of population variability, and performance for chemicals with complex toxicokinetics. Broader evaluation across additional chemical categories and toxicological endpoints will be essential to more confidently establish the reliability and regulatory applicability of NAM-based PODs.

### 6.2 Perspectives

**FIGURE 2:**
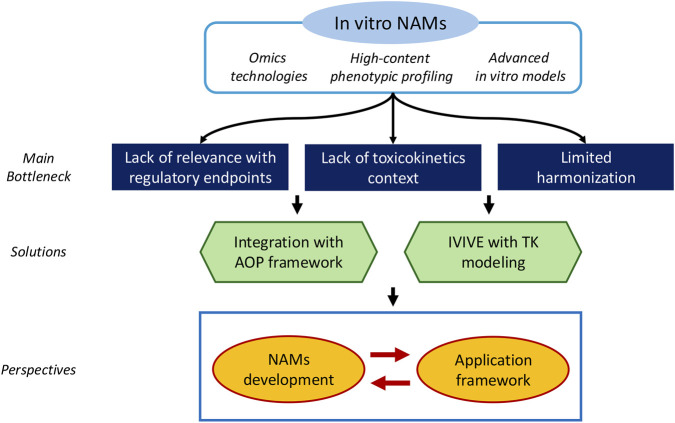
Summary of the study.

Advancing the use of *in vitro* NAMs in next-generation risk assessment will require not only continued development of new and improved assays but also the establishment of robust frameworks and decision structures that enable effective use of the NAMs that already exist. Although much of the current research landscape is heavily weighted toward NAMs development, technological progress alone is insufficient. It is equally important to apply available NAMs in real-world decision contexts—acknowledging their inherent limitations while still extracting meaningful insights—and to use the lessons learned from these applications to guide future NAM innovation. This bidirectional, iterative process can create a positive feedback loop in which NAM use informs NAM development, ensuring that new assays evolve in alignment with practical regulatory needs (Figure 2). Chemicals differ widely in the breadth and depth of NAM data available for them. While some chemicals are supported by rich datasets across multiple platforms, many others possess only minimal *in vitro* information or even just *in silico* predictions. For example, although we emphasize the need to consider toxicokinetic context when interpreting *in vitro* NAM data within the NGRA framework, the reality is that most environmental chemicals lack even the most basic toxicokinetic information (Beal, 2021). In such cases, the use of nominal concentrations as a conservative default assumption becomes practically unavoidable and can serve as a protective interim approach until more refined toxicokinetic or IVIVE data become available. This reflects a broader point in our perspective: while the conceptual NGRA framework is becoming increasingly mature, practical implementation must account for substantial variability in data availability across chemicals. As a result, a tiered, data-dependent NAM utilization framework is essential. Such a framework should allow decision-makers to adapt the level of analysis to the data available—using rapid screening assays when information is sparse, incorporating mechanistic or pathway-level NAMs as more data accumulate, and integrating multiple evidence streams when higher confidence is required. Rather than relying on any single NAM as a standalone decision tool, future efforts should focus on building evidence-integration systems that synthesize diverse lines of information, including mechanistic NAM outputs, toxicokinetic modeling, read-across analogs, and exposure considerations (Weed, 2005). Structured integration will allow even incomplete or heterogeneous datasets to support more transparent and consistent decision-making. Ultimately, progress in NGRA will depend not solely on the sophistication of NAM technologies but on the development of a flexible, adaptive decision framework that can incorporate both current and emerging tools. By fostering a dynamic interaction between NAM development and NAM application, the field can move toward a more coherent, resilient, and scientifically grounded approach to chemical risk assessment.
